# Community Engagement in the Fight Against COVID-19: Knowledge, Attitude, and Prevention Practices Among Dire Dawa Residents, Eastern Ethiopia

**DOI:** 10.3389/fpubh.2021.753867

**Published:** 2021-11-30

**Authors:** Abdurezak Umer, Kedir Abdella, Yared Tekle, Addisalem Debebe, Tsegahun Manyazewal, Mohammed Yuya, Hussen Mohammed

**Affiliations:** ^1^Department of Public Health, College of Medicine and Health Sciences, Dire Dawa University, Dire Dawa, Ethiopia; ^2^Center for Innovative Drug Development and Therapeutic Trials for Africa (CDT-Africa), College of Health Sciences, Addis Ababa University, Addis Ababa, Ethiopia; ^3^School of Public Health, College of Health and Medical Sciences, Haramaya University, Harar, Ethiopia

**Keywords:** coronavirus disease 2019 (COVID-19), SARS-CoV-2, knowledge, attitude, practice, community

## Abstract

**Background:** There is a global concern that coronavirus disease 2019 (COVID-19) cannot be addressed without the integration and active engagement of communities. We aimed to investigate the knowledge, attitude, and practices (KAP) of the residents in Eastern Ethiopia toward COVID-19.

**Method:** A community-based cross-sectional study was conducted on May 1–30, 2020 among the residents of Dire Dawa, Eastern Ethiopia. Data were collected using a structured questionnaire on the awareness, knowledge, attitudes, and preventive practices toward COVID-19. We used random sampling to select the participants. Data was entered into the statistics and data (STATA) version for data cleaning and analysis. Binary logistic regression models with 95% CI were used to conduct bivariable [crude odds ratio (COR)] and multivariable [adjusted odds ratio (AOR)] analyses.

**Result:** A total of 415 community residents responded to the questionnaire. Of those participants, 52.5% (95% CI 47.7–57) had adequate knowledge, 31.6% (95% CI 27–36) had favorable attitudes, and 49% (95% CI 45–50) had good practice toward the precautionary measures of COVID-19. Adequate knowledge had a significant association with urban residence (AOR = 5, 95% CI 3.1–8.4) and literacy (AOR = 3.1, 95% CI 1.5–6.7). Good preventions practices had a significant association with place of residence (AOR = 4.1, 95% CI 2.3–7.2), literacy (AOR = 2.9, 95% CI: 1,2–7.4), adequate knowledge (AOR = 3.5, 95% CI 2.3–5.8), and favorable attitude (AOR = 2.3, 95% CI 1.4–3.8) about the disease.

**Conclusion:** The overall COVID-19-related KAP was inadequate in the majority of the residents of Dire Dawa that occupy irregular migration flows. These call for robust community-centered behavioral communication strategies that could bridge the gaps and help prevent and control COVID-19 and other future pandemics in their community.

## Introduction

Coronavirus disease 2019 continues to be a global pandemic and public health crisis. More than 200 million people were infected by the disease and 2% of these people died, globally (1). Ethiopia reported its first coronavirus disease 2019 (COVID-19) case on March 13, 2020; since then, the disease has spread over all parts of the country and infected over 300,000 people, of whom 1.59% died (1). The majority of the secondary cases reported in Ethiopia were acquired through community transmission (2). Currently, there are some specific antiviral treatments recommended for COVID-19 infection (3). Individuals with COVID-19 often receive medical care to relieve symptoms. Recently, a high number of COVID-19 cases were reported in Ethiopian communities, even though the Federal Government of Ethiopia and its national regional states and city administrations took steps to prevent the spread of the disease (4). The outbreak still has the potential for greater loss of life in Ethiopia if the community is unable to shape their regular behavioral and sociocultural norms that would favor the spread of the disease. The knowledge, attitude, and practices (KAP) of the community toward COVID-19 have a significant impact on the battle against the disease. Previous studies conducted elsewhere around KAP documented cognitive factors as major drivers of behavioral change (5, 6) and the KAP of the disease was the major behavioral determinant that directly influenced the activities of human beings, behaviors, understanding, and habits of a given community (7). There have been some studies conducted in Ethiopia to know how the KAPs toward COVID-19 at the community level influenced the transmissions of the disease (8, 9); however, their primary focus had been on the general knowledge of COVID-19 or knowledge that the general public can have along with the misconception of COVID-19.

The Dire Dawa administration is an industrial hub, in which cars, railways, and airline transportations are available, and a border with Djibouti that favors the transmission of COVID-19. There has been no research conducted in Dire Dawa to know the KAP of the community toward COVID-19 and this has been a missed opportunity to mitigate the disease in its earliest stages. In the absence of adequate treatments and vaccines in the place, the strict implementation of disease prevention and control measures, and harnessing the awareness of communities toward these measures are the most powerful tools that local governments need to pursue. In this case, it is necessary to assess the KAPs, beliefs, rumors, and misconceptions of a given community to know the appropriate interventions.

Therefore, the present study aimed to investigate the current KAP of the residents in the Dire Dawa administration, Eastern Ethiopia, toward COVID-19.

## Methods

### Study Design and Study Area

A community-based, cross-sectional study was conducted in the Dire Dawa Administration. The Dire Dawa administration is situated in the Eastern part of Ethiopia, 315 km from Addis Ababa, the capital city of Ethiopia. The Dire Dawa administration has different sorts of transportations from land, railway, and an international airport, and it is only 300 km away from Djibouti, thus is susceptible to cross-border transmission of COVID-19. The administration has two government hospitals and 15 health centers that provide healthcare services to more than 500,000 of the population (10).

## Participants

The source population was all the residents of the Dire Dawa administration, and the study population was all selected adults in the selected kebeles (lowest administrative unit) in the urban and rural kebeles. The following inclusion criteria were applied in this study: adults (i.e., older than 18 years of age) of any sex, formal residents of Dire Dawa, and willing to participate and sign the informed consent of the study.

The sample size estimation was calculated using a single population formula with a 5% margin of error and 95% confidence level. We considered that 50% of the population have adequate KAP based on the sample collection procedure at the times that there is no study to refer to (11). With this, a minimum of 384 participants was required. A further 10% was added to account for non-respondents and this increased the sample size to 422.

Random sampling was used to select the study participants. In the Dire Dawa administration, there are urban and rural kebeles (lowest administration unit). Three urban and six rural kebeles were selected using simple random sampling (SRS). Finally, the sampling with population proportional to the size was calculated for the study participants at each selected kebeles to attain the total sample size by using the proportionate allocation formula.

### Data Collection

The data were obtained from the consenting participants through face-to-face interviews using a structured questionnaire on the KAP toward COVID-19. The questionnaire was adopted from previous studies (12, 13) and pre-tested. It had five distinct sections that deal with sociodemographic data, KAP, awareness toward the disease, and the belief, rumors, and stigma associated with COVID-19 in the community. The data were collected from May 1–30, 2020.

### Data Quality Control

On the survey instrument, omit or skip patterns were utilized to properly direct respondents to answer items that are relevant to them. A codebook was employed to input and organize the data appropriately. Data verification processes, including the spot-checking of the questionnaire at the data-gathering sites, were implemented to ensure the accuracy of the row data. The questionnaire was pre-tested on 5% of the total sample size.

### Data Analysis

The data were double-entered in Epi-Info™ (version 3.5.1, CDC, Atlanta, Georgia, United States), and analyzed by Stata 14 (College Station, Texas, United States). A Kolmogorov-Smirnov test was used to check for the normal distribution of data. The reliability of the questionnaire was tested using Cronbach's alpha. Binary logistic regression models with 95% CI were used to conduct bivariable [crude odds ratio (COR)] and multivariable analysis [adjusted odds ratio (AOR)]. From the multivariable analysis (AOR), variables significantly associated with *p*-value < 0.05 were declared as factors for good prevention practices.

### Outcome Variables

The knowledge of the respondents about COVID-19 was assessed using a 31-item questionnaire developed by Zhong et al. and adopted by other similar studies (12, 13). The questionnaire included the clinical characteristics of the disease (i.e., primary symptoms, availability and effectiveness of treatment, and severity), addressed transmission (i.e., infection through contact with animals and transmission through respiratory droplets), and prevention and control (i.e., wearing medical masks for prevention). All respondents could respond with “Yes,” “No,” or “Don't know.” The knowledge scores were calculated by assigning one point to each correct question, and an aggregate score was calculated (range 0–21). It is measured by calculating the mean score of the 21 items. This was categorized as knowledgeable, if a participant scored ≥ the mean score of the correctly answered questions, or not knowledgeable, if the participant scored < the mean score of the correctly answered questions

To measure attitudes related to COVID-19, we examined the perceived risk of COVID-19 infection comprising perceived susceptibility, which signifies the beliefs of an individual about their possibility of infection, and perceived severity of the infection. The respondents answered how dangerous they think COVID-19 is, how they describe their risk of infection with COVID-19, and how effective the state of emergency was to control the spread of COVID-19. The responses were rated on a 5-point Likert-type scale, with “1 = very low, 3 = neither low nor high, and 5 = very high” which is finally merged.

The prevention practice behaviors were measured using three items that covered the following two categories: (1) preventive measures (i.e., wearing facial masks and practicing hand hygiene) and social distancing (i.e., avoiding crowded places). The respondents self-reported the frequency of the practices they performed during the previous week at the time of the survey, using yes “1” and no “0”).

### Ethical Consideration

The study protocol was reviewed and approved by the *ad-hoc* Committee for Ethical Review of Human Study of the Dire Dawa University. A letter of support was written to the concerned body. Participation was voluntary and each participant was asked to give consent after a clear explanation of the research objectives. The participants were assured that all responses would remain confidential. The right to withdraw from the study was respected.

## Results

### Socio-Demographic Characteristics

Out of the 422 community residents that the study reached, 415 participants consented and responded to the questionnaire, with a respondent rate of 98%. For their place of residence, 371 (89.4%) were from urban areas. The mean age of the participants was 32.83 ± 8.78 SD. The majority of the participants were male [268 (64.6%)] and government employees [113 (27.2%)]. Regarding their educational status, 66 (15.9%) cannot read and write and 158 (38.1%) have attended college and above (Table 1).

**Table 1:** Demographic characteristic of study participants on the assessment of the risk of COVID-19 among Dire Dawa administration residents, Eastern Ethiopia, May 2020 (*N* = 415).

**Characteristics**		**Number**	**Percentage**
Place of residence	Urban	271	65.3
	Rural	144	34.7
Gender of participants	Male	268	64.6
	Female	147	35.4
Age of the participants	18–29	163	39.3
	30–64	249	60
	65 and above	3	–
Marital status	Single	117	28.2
	Married	273	65.8
	Divorced	15	3.6
	Widowed	10	2.4
Occupation	Driver	85	20.5
	Merchant	70	16.9
	Government employee	114	27.5
	Daily labor	79	19.1
	House-wife	59	14.2
	Student	8	–
Educational status	Illiterate	66	15.9
	Primary	83	20
	Secondary	108	26
	college and above	158	38.1
Monthly income	<21.5 USD	29	7
	21.6–64.5 USD	204	49.1
	>64.5 USD^*^	182	43.9

* *USD, United State Dollar*.

### Awareness on COVID-19

**Figure 1:**
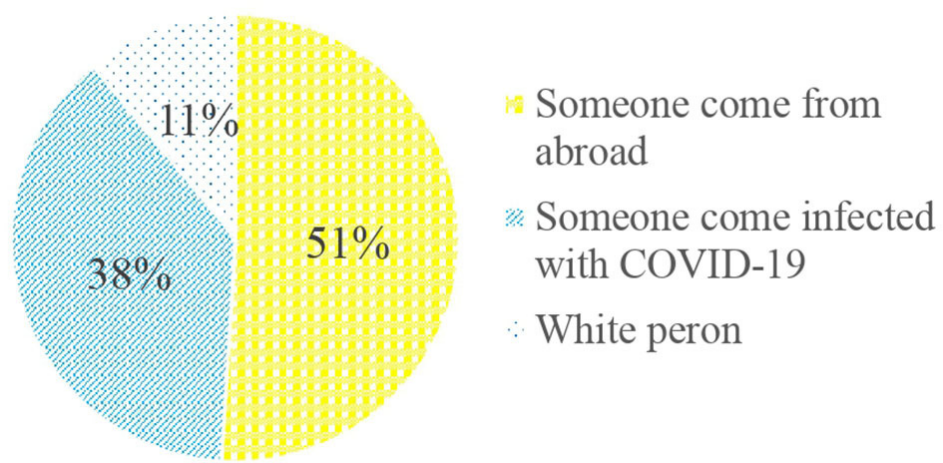
Stigmatized group during the COVID-19 pandemic among the Dire Dawa Administration residents, Eastern Ethiopia, September 2020.

Almost all the participants (99.9%) have heard about COVID-19, 356 (85.8%) were aware that COVID-19 is a virus that causes a disease, and 52 (12.5%) perceived COVID-19 as futile propaganda from the government. Regarding the source of information, 359 (86.5%) of the participants have heard of the disease from television, with 273 (65.8%) and 88 (21.2%) perceiving television and social media as the ideal source of information, respectively. In the study, 228 (54.9%) of the participants reported that there is a stigma toward COVID-19 (Figure 1).

### Knowledge on COVID-19

A total of 21 questions were used to measure the knowledge of the study participants toward COVID-19 where the mean knowledge score was 14.29 (SD= 4.7, range 3–26). Two hundred forty-six (59.3%) of the participants knew that people who had contact with an infected person should be immediately isolated for 14 days as per the national guideline. Regarding social interaction, 395 (95.2%) of the participants perceived that shaking hands and hugging may result in the contract of COVID-19. Regarding preventive measures, 360 (86.7%) and 267 (64.3%) of the participants perceived that washing hands with soap and wearing a mask in crowded areas, respectively, prevent COVID-19 transmission (Table 2).

**Table 2:** Frequency of respondent's knowledge on coronavirus, Dire Dawa administration, Eastern Ethiopia, May 2020 (*N* = 415).

**Questions to assess the knowledge of study participants**	**Yes**		**No**	
	***n***	**%**	***n***	**%**
Symptoms of COVID-19 are fever, fatigue, dry cough, headache	372	90.1	43	9.9
Knowledge on availability of cure for COVID-19	294	70.8	121	29.2
Blood transmission will result in infection of COVID-19	14	3.4	401	96.6
Droplet from infected person may result in infection of COVID-19	197	47.5	218	52.5
COVID-19 is transmitted from one person to other through airborne	261	62.9	154	37.1
COVID-19 is transmitted through sexual intercourse	219	52.8	196	47.2
COVID-19 is transmitted touching contaminated object	310	74.7	105	25.3
Eating or touching infected wild animals will result in infection of COVID-19	22	3.3	393	94.7
Handshaking and hugging will transmit COVID-19	395	95.2	20	4.8
Do you think children under five are the riskiest group?	202	48.7	213	51.3
Do you think adolescents and adults are the riskiest groups?	175	42.2	240	57.8
Do you know individuals exposed to or travel history should be isolated for 14 days?	246	59.3	169	40.7
Do you think older people are the riskiest group?	353	85.1	62	14.9
Do you think pregnant women are the riskiest group?	212	51.1	203	48.9
Do you think health professionals are the riskiest group?	193	46.5	222	53.5
Do you think COVID-19 could be prevented by keeping social distance?	383	92.3	32	7.7
Do you think washing hands regularly can prevent COVID-19?	360	86.7	55	13.3
Do you think covering mouse and nose while sneezing and going out to crowded place will	306	73.7	109	26.3
Avoiding close contact with an individual who has a fever?	278	67.0	137	33.0
Wearing mask will prevent infection of COVID-19	267	64.3	148	35.7
Cooking raw food and avoiding contact with an animal will prevent infection of COVID-19	135	62.5	280	67.5

### Attitudes Toward COVID-19

A total of four questions were used to assess the attitudes of the participants toward COVID-19. These included questions if they perceive COVID-19 as a serious disease, perceive the State of Emergency of the government as effective in controlling COVID-19, if complete lockdown helps to control COVID-19 in their community, and if they perceive that they will become infected with COVID-19 without their personal preventive measures. With these, 271 (65.3%) of the participants perceived COVID-19 as a very serious disease; 144 (34.7%) perceived that the State of Emergency would help prevent the transmission of the disease; 180 (43.4%) thought the State of Emergency was not implemented thoroughly as planned. Additionally, 300 (72.3%) of the participants thought that the lockdown was an ideal measure to contain the disease in their community (Table 3).

**Table 3:** Frequency of respondent's attitudes on coronavirus, Dire Dawa administration, Eastern Ethiopia, May, 2020 (*N* = 415).

**Attitudes question**	**Very high** **(very good)**		**High (good)**		**Not high (normal)**	
	***n***	**%**	***n***	**%**	***n***	**%**
How danger is corona virus	271	65.3	131	31.6	13	3.1
How effective state of emergency is control and prevention of COVID-19 infection	144	34.7	180	43.3	91	22
How do you describe your perception toward lockdown during COVID-19 pandemic	99	23.9	300	72.3	16	3.8
How do you describe your risk of infection with COVID-19	115	27.7	177	42.7	123	29.6

### Practice on COVID-19 Prevention and Control

A total of eight questions were used to assess the COVID-19 prevention practices of the participants, with 346 (83.4%) participants saying that they stopped going to crowded places and 230 (55.4%) used face masks if they had to go to a crowded place. Similarly, 301 (72.5%) of the participants perceived that they keep social distancing, 399 (96.1%) wash their hands with soap frequently, and 229 (55.2%) stopped shaking hands (Table 4).

**Table 4:** Frequency of respondent's practice toward COVID-19, Dire Dawa administration residents, Eastern Ethiopia, May 2020 (*N* = 415).

**Practice related questions**	**Yes**		**No**	
	***n***	**%**	***n***	**%**
You avoid going to a crowded place?	346	83.4	69	16.6
Do you practice social distancing?	301	72.5	114	27.5
Do use a mask whenever you go out of your home?	230	55.4	185	44.6
Do you wash your hand with soap on regular basis?	399	96.1	16	3.9
Do you wash your hand with sanitizer on regular basis?	303	73.0	112	27.0
Do you stay at home when you are sick?	88	21.2	327	78.8
Do you cover your mouth and nose with an elbow while sneezing and coughing?	257	61.9	158	38.1
Do you avoid handshaking?	229	55.2	186	44.8
Do you practice respiratory hygiene daily?	300	72.3	115	27.7

### Factors Associated With Knowledge About COVID-19

**Figure 2:**
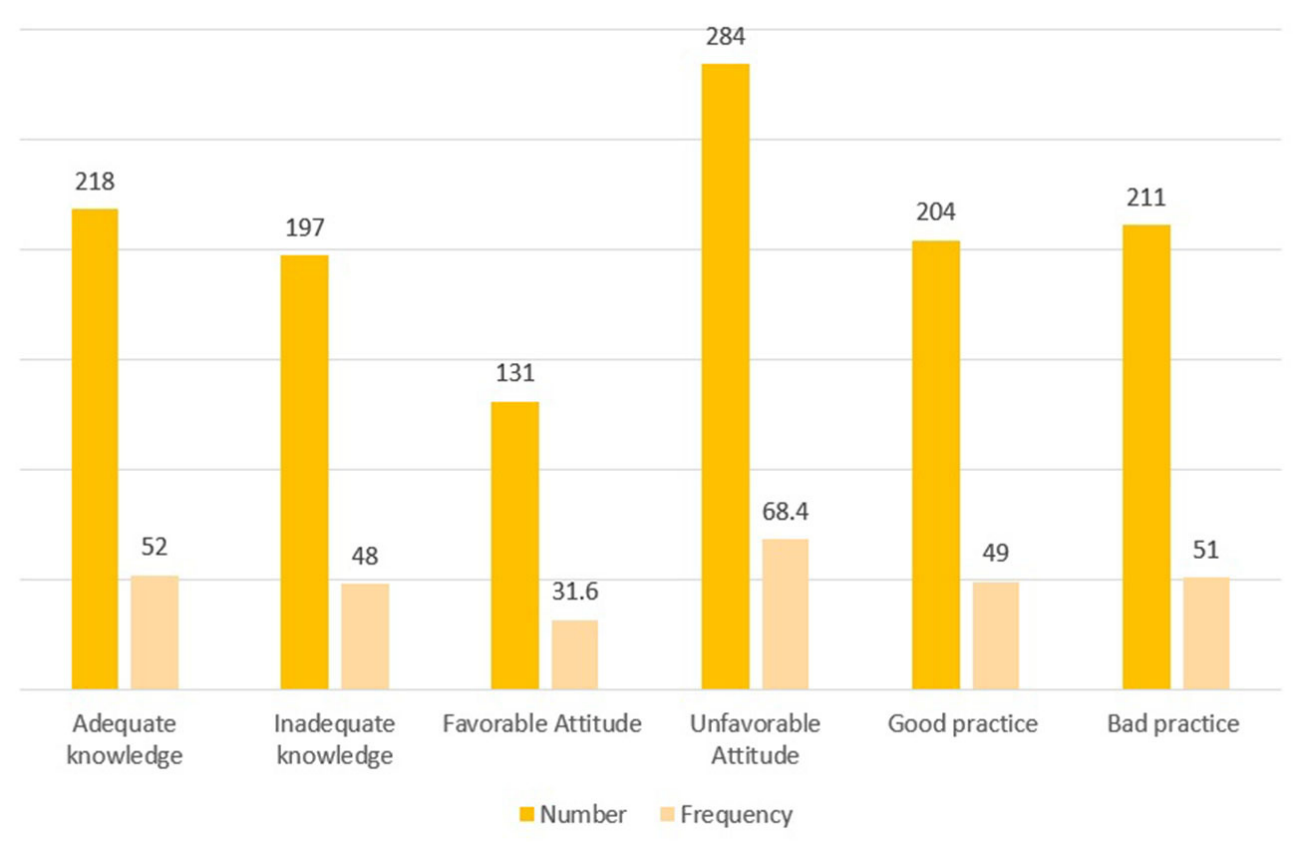
Distribution of knowledge, attitude, and practice toward COVID-19 among the Dire Dawa Administration residents, Eastern Ethiopia, September 2020.

Variables such as place of residence, sex, educational level, monthly income, and occupation of participants had a *p*-value of < 0.2 in the bivariate analysis and were associated with their knowledge about COVID-19. These variables were entered into a multivariate binary logistic regression analysis model. The multivariate analysis showed that the urban residents were five times [AOR 5, 95% CI (3.1.−8.4)] more likely to have adequate knowledge, attitude and practice of study participants is shown in Figure 2 compared with the rural residents. Similarly, the participants who had formal education from registered institutions were 3.1 times [AOR 3.1, CI (1.5–6.7)] more likely to have adequate knowledge than those without formal education (Table 5).

**Table 5:** Factors associated with knowledge toward COVID-19 among Dire Dawa administration residents, Eastern Ethiopia May 2020.

**Variables**		**Knowledge**		**COR 95% CI**	**AOR 95% CI**
		**Adequate *N* (%)**	**Not-adequate *N* (%)**		
Place of residence	Rural	41 (28.4)	103 (71.5)	1	1
	Urban	94 (34.7)	177 (65.4)	4.7 (3.04–7.3)***	5.0 (3.1–8.4)***
Sex	Female	61 (41.50)	86 (58.5)	1	1
	Male	157 (58.58)	111 (41.42)	2.0 (1.3–3.0)**	1.8 (1.1–3.2)*
Educational status	Illiterate	13 (19.7)	53 (80.3)	1	1
	Educated	205 (58.7)	144 (41.3)	5.8 (3.1–11.03)***	3.1 (1.5–6.7)***
Occupation	House wife	20 (33.9)	39 (66.10)	1	1
	Government employee	73 (64.04)	41 (35.96)	3.4 (1.7–6.7)	1.0 (0.3–2.4)
	Driver	74 (55.29)	38 (44.71)	2.4 (1.2–4.7)*	0.8 (0.3–2.3)
	Merchant	40 (57.12)	30 (42.86)	2.6 (1.2–5.3)*	0.9 (0.3–2.6)
	Daily labor	33 (41.8)	46 (58.2)	1.8−6.7	1.5 (0.6–3.8)
	Student	5	3	3.2 (0.7–15.0)	2.1 (0.3–13.6)
Average monthly income birr	<21.5 USD	13 (44.83)	16 (55.17)	1	1
	21.6–64.5 USD	92 (45.1)	112 (54.9)	1.0 (0.46–2.2)	1.1 (0.3–3.3)
	>64.5 USD*	113 (62.1)	69 (37.9)	2.01 (0.9–4.4)	1.6 (0.4–5.6)

*USD, United State Dollar; COR, Crude OR; AOR, Adjusted OR; CI, Confidence Interval. Superscript indicates statistical significance. P-value ≤ 0.05, **P-value < 0.01, ***P-value < 0.001.

### Factor Associated With COVID-19 Prevention Practice

Variables such as level of knowledge, place of residents, sex, educational status, income, and occupation of the participants were significantly associated with COVID-19 prevention practice in the bivariate analysis. In the multivariate logistic regression model, level of knowledge, attitude, place of residents, sex, educational status, and occupation are significantly associated with COVID-19 prevention practices. Participants who had adequate knowledge were 4.9 times more likely to practice COVID-19 preventive measures compared with those who do not have adequate knowledge. The participants who had a favorable attitude toward COVID-19 were 2-fold likely to practice COVID-19 prevention measures. Men were 60% less likely to practice COVID-19 prevention measures as compared with women (Table 6).

**Table 6:** Factors associated with practice toward COVID-19 among Dire Dawa administration residents, Eastern Ethiopia, May 2020.

**Variables**		**Practice**		**COR 95% CI**	**AOR 95% CI**
		**Good practice *N* (%)**	**Bad practice *N* (%)**		
Knowledge status	Not-Adequate	55 (27.9)	142 (72.1)	1	1
	Adequate	152 (69.7)	66 (30.3)	5.9 (3.9–9.1)***	3.5 (2.1–5.8)***
Attitude status	Unfavorable	123 (43.3)	161 (56.7)	1	1
	Favorable	84 (64.1)	47 (35.9)	2.3 (1.5–3.5)***	2.3 (1.3–3.8)**
Place of residence	Rural	33 (22.9)	111 (77.1)	1	1
	Urban	174 (64.2)	97 (35.8)	6.0 (3.8-9.5)***	4.1 (2.3-7.2)
Sex	Female	52 (35.3)	95 (64.6)	1	1
	Male	155 (57.8)	113 (42.1)	2.5 (1.6–3.7)***	2.5 (1.3–4.6)*
Educational status	Illiterate	8 (12.1)	58 (87.9)	1	1
	Educated	199 (57.1)	158 (42.9)	9.6 (4.5–2.7)***	2.9 (1.2–7.4)**
Occupation	House wife	16 (27.1)	43 (72.9)	1	1
	Driver	49 (57.7)	36 (42.3)	3.6 (1.7–7.4)***	1.01 (0.3–3.0)
	Government employee	78 (68.4)	36 (31.9)	5.8 (2.9–11.6)	1.3 (0.4–4.0)
	Merchant	35 (50)	35 (50)	2.6 (1.2–5.6)	0.8 (0.2–2.5)
	Daily labor	23 (11.1)	56 (26.9)	1.1 (0.5–2.3)	0.5 (0.2–1.6)
	Student	6	2	8.0 (1.4–44.1)	5.8 (0.6–56.5)
Monthly income	<21.5 USD	12 (41.3)	17 (58.62)	1	1
	21.6–64.5 USD	116 (56.9)	88 (43.1)	1.1 (0.5–2.3)	1.4 (0.4–4.9)
	>64.5 USD*	107 (58.8)	75 (41.2)	2.02 (1.9–4.4)*	1.1 (0.2–4.6)

*USD, United State Dollar; COR, Crude OR; AOR, Adjusted OR; CI, Confidence Interval. Superscript indicates statistical significance. P-value ≤ 0.05, **P-value < 0.01, ***P-value < 0.001.

## Discussion

One of the main goals of this study was to investigate the KAP of Dire Dawa residents toward COVID-19. This issue is critical for research to identify the necessary intervention based on the gaps we identified. The study revealed that close to half of the study participants had inadequate knowledge regarding COVID-19; similarly, two-third of the participants had unfavorable attitudes toward COVID-19, and about half had poor practice toward COVID-19 preventive measures.

More than half (54%) of the participants in this study perceived that there was a COVID-19-related stigma in their community; this finding was lower as compared with the finding from a study conducted in Jima Zone which reported 83.3% of stigma toward the disease (13). The observed difference may be due to differences in the study period or demographic characteristics of the study participants. This finding showed that there is an urgent need for an intervention to handle stigma. The WHO emphasized that stigma during such an outbreak demands an urgent intervention (14).

The study revealed that 52% of the study participants have adequate knowledge of COVID-19, and the finding was lower than a previous national-level study in Ethiopia that reported an adequate knowledge of 90% among the participants (15). A possible explanation for this discrepancy could be that the participants of the study were interviewed virtually and such participants could have higher education levels than the current study. Inversely, the current study yielded a higher value compared with a previous study conducted in the Mizan Tepi community, Southern Ethiopia, where 43% of the study participants had adequate knowledge about COVID-19 (8). Comparing our findings with studies conducted outside of Ethiopia, our findings were higher than a study conducted in Bangladesh in which 65% of the participants had inadequate knowledge about COVID-19 (16). This discrepancy may be attributed to the difference in the socio-demographic factors of the study participants.

In this study, 86% of the knowledge of the participants about COVID-19 came from the television as the main source of information, which was in agreement with a previous study conducted in Nigeria (17) but in contrast to a study conducted in Egypt that reported social media as the main source of information (18).

The findings of this study showed that the majority (92%) of the study participants reported that they had regularly used social distancing while some (83 %) of them used frequent hand washing (soap and sanitizer), which are the most effective ways to prevent oneself from COVID-19. A similar finding was reported from a previous study performed in the Jima University teaching hospital in Ethiopia. Similarly, a study conducted in Egypt reported social distancing and proper handwashing as the main ways to protect oneself against coronavirus (19). Furthermore, a study conducted in Italy also considered social distancing as the main defense against coronavirus (20). This study finding showed that the participants who had less educational level were less knowledgeable about coronavirus transmission and prevention. Similar to a study from Southern Ethiopia (21), Egypt (18), and Saudi Arabia (22). However, a study conducted in the Jima University teaching hospital found that education is not associated with the knowledge status of COVID-19 (13).

The findings of this study showed that about one-third of the study participants had a favorable attitude toward coronavirus prevention policy and risk level; this is in line with the study conducted by Bekele D et al. at the national level in Ethiopia (13). However, this result differs from the high level of favorable attitude (92.%) found in southern Ethiopian residents (23). Regarding the trust of the population in the effectiveness of government policies in the prevention and control of the coronavirus, only one-third of the study participants had a favorable attitude; this is similar to a study conducted in Egypt (24) and Nigeria (25), where the participants believe that it was a biological weapon; this may result in valuing the preventive methods less, which is dangerous in controlling the fastest spreading coronavirus.

The findings of this study showed that about half of the study participants had poor practice toward the prevention of coronavirus. This is in line with a study conducted in Addis Zemen Hospital, Northwest Ethiopia (26). However, this finding is higher than the study conducted in Bangladesh where only 19% of the respondents had poor practice toward coronavirus prevention methods (27). Our study revealed that most of the study participants had malpractice toward COVID-19 prevention measures despite their adequate knowledge. For instance, only 61% of participants used a mask daily, although the majority of the respondents reported that they knew the importance of masks. This finding shows that it takes repeated health education to change the behavior of an individual. This study finding showed that being rural residents, the level of education in terms of sociodemographic was associated with the poor practice of coronavirus prevention methods. This finding was consistent with studies done in southern Ethiopia (8), Egypt (24), China (28), and Malaysia (29).

Overall, among the studied demographic characteristics, the place of residence, sex, and education had a significant association with the knowledge and practice of COVID-19 transmission and preventive measures. However, the attitudes of the participants did not show significant association with the actual practice. In comparison to this finding, a couple of studies reported that place of residence, educational level, and sex were significantly associated with knowledge status (18, 23, 28), and another study showed the association of sex and educational level (22). Furthermore, previous studies have shown that the place of residents and education status were significantly associated with COVID-19 prevention practices (26). Additionally, significant associations were found between knowledge status and attitude (29). This difference may be due to differences in the population characteristics. In certain areas, there are low levels of early adoption of the new practices while in other geographic areas, people adopt new practices early. In our study, males had a higher level of knowledge and practice toward transmission and prevention methods. This could be explained by the responding tendency of the participants and the statistical allocation of the study participants.

The low level of preventive practices among the participants of this study may reflect the unstable conditions and economic status of the individuals and country as a result of the current coronavirus pandemic. Many government and private-sector employees have lost their jobs nationally and internationally (30, 31), and the pandemic interrupts the healthcare services for other disease conditions (32). The main concern of these people is to provide their families with daily food requirements. As a result, the financial shortage has affected the knowledge and preventive behavior of people against COVID-19. The use of information technology, including digital health solutions, may help build the KAP potential of the community through training, dissemination of up-to-date information about the disease, and exchange of information that can combat misinformation (33–35).

In general, although the COVID-19 KAPs studies in Ethiopia are limited, there is a significant variation in the KAP between the studies, which could be attributed to the differences in the study areas, study population, and the study design followed.

## Conclusion

Our findings revealed that there are significant knowledge gaps, unfavorable attitudes, and poor practices toward COVID-19 among the Dire Dawa administration residents in Eastern Ethiopia. Predictor variables including place of residence, sex, and educational level were significantly associated with the knowledge scores. Predictive variables including knowledge score, attitude, place of residence, and educational level were significantly associated with the practice levels. These call for robust community-centered behavioral communication strategies that could bridge the gaps and help prevent and control COVID-19 and other future pandemics in their community.

## Data Availability Statement

The original contributions presented in the study are included in the article/supplementary material, further inquiries can be directed to the corresponding author/s.

## Ethics Statement

The studies involving human participants were reviewed and approved by Dire Dawa University office of the vice president by *ad-hoc* Committee for Ethical Review of Human Study. The patients/participants provided their written informed consent to participate in this study.

## Author Contributions

AU participated in all phases of preparation starting from the inception of the project, collection of data, analysis and interpretation of results, and writing of the manuscript. HM contributed to the study design and checklist development during the data collection and writing of the manuscript. TM and MY contributed to the writing of the manuscript. AD contributed to the data collection and analysis. YT participated in the questionnaire development and analysis and manuscript preparation. KA participated in the questionnaire development and data collection. All authors read and approved the final manuscript.

## Funding

This work was funded by the Dire Dawa University.

## Conflict of Interest

The authors declare that the research was conducted in the absence of any commercial or financial relationships that could be construed as a potential conflict of interest.

## Publisher's Note

All claims expressed in this article are solely those of the authors and do not necessarily represent those of their affiliated organizations, or those of the publisher, the editors and the reviewers. Any product that may be evaluated in this article, or claim that may be made by its manufacturer, is not guaranteed or endorsed by the publisher.
